# Rural exposure during medical education and student preference for future practice location - a case of Botswana

**DOI:** 10.4102/phcfm.v8i1.1039

**Published:** 2016-06-10

**Authors:** Tonya Arscott-Mills, Poloko Kebaabetswe, Gothusang Tawana, Deogratias O. Mbuka, Orabile Makgabana-Dintwa, Kagiso Sebina, Masego Kebaeste, Lucky Mokgatlhe, Oathokwa Nkomazana

**Affiliations:** 1 Botswana-UPenn Partnership, Botswana; 2 Department of Paediatrics, Perelman School of Medicine, University of Pennsylvania, USA; 3 Department of Paediatrics, University of Botswana, Faculty of Medicine, Botswana; 4 Medical Education Partnership Initiative, University of Botswana, Faculty of Medicine, Botswana; 5 Department of Family Medicine, University of Botswana, Botswana; 6 Department of Surgery, University of Botswana Faculty of Medicine, Botswana

## Abstract

**Background:**

Botswana’s medical school graduated its first class in 2014. Given the importance of attracting doctors to rural areas the school incorporated rural exposure throughout its curriculum.

**Aim:**

This study explored the impact of rural training on students’ attitudes towards rural practice.

**Setting:**

The University of Botswana family medicine rural training sites, Maun and Mahalapye.

**Methods:**

The study used a mixed-methods design. After rural family medicine rotations, third- and fifth-year students were invited to complete a questionnaire and semi-structured interview. Data were analysed using descriptive statistics and thematic analysis.

**Results:**

The thirty-six participants’ age averaged 23 years and 48.6% were male. Thirty-three desired urban practice in a public institution or university. Rural training did not influence preferred future practice location. Most desired specialty training outside Botswana but planned to practice in Botswana. Professional stagnation, isolation, poorly functioning health facilities, dysfunctional referral systems, and perceived lack of learning opportunities were barriers to rural practice. Lack of recreation and poor infrastructure were personal barriers. Many appreciated the diversity of practice and supportive staff seen in rural practice. Several considered monetary compensation as an enticement for rural practice. Only those with a rural background perceived proximity to family as an incentive to rural practice.

**Conclusion:**

The majority of those interviewed plan to practice in urban Botswana, however, they did identify factors that, if addressed, may increase rural practice in the future. Establishing systems to facilitate professional development, strengthening specialists support, and deploying doctors near their home towns are strategies that may improve retention of doctors in rural areas.

## Introduction

Globally health care workforce shortages are a major problem in rural areas. The World Health Report 2006 established that 57 countries had ‘critical shortages’ of health workers; 36 of those countries were in sub-Saharan Africa.^1^ In addition, of the few health care workers available, the majority are in urban areas.^1^ Human resources for health are the backbone of service delivery in the health sector and vital for improving health. Training for rural practice has become critically important in the context of this continuing serious shortage of health care workers in rural areas.^2^ In 2010, the World Health Organization published guidelines to assist ministries of health in developing policies to attract and retain workers in underserved areas.

Research suggests that there are many factors affecting recruiting and retaining workers in rural areas and they include 1) factors associated with the workers background such as coming from a rural area or more disadvantaged background^3,4,5^; 2) factors related to the educational setting and exposure during training, both duration of exposure, and the quality of the experience^5,6^; and 3) factors that are related to the environment and job itself in these locations, such as a resources, salary, educational opportunities for children, and recreational activities.^2,4,7,8,9^ A recently published study from Botswana supports that the cause of health worker shortages in primary care and rural areas in Botswana is multifactorial.^10^

Creating an appropriately skilled, highly motivated, client-focused health workforce is critical for Botswana to attain its ambition of ensuring an enabling environment, in which all people living in Botswana have the opportunity to reach and maintain the highest attainable level of health.^11^ Historically the training of medical doctors in Botswana has been outside of the country.^11^ Few of those trained externally returned and the country has relied on a foreign workforce.^11,12,13^ In an effort to minimise reliance on a foreign workforce and address retention issues the University of Botswana Faculty of Medicine (UBFoM) was established in 1998 (as the School of Medicine) with its first enrolment in 2009. The UBFoM MBBS curriculum aims to produce appropriately skilled generalist doctors who can function within primary care providing quality care and access to health care.^12^ In this effort it has incorporated exposure to rural health care delivery throughout the 5-year curriculum.

Although factors associated with recruiting and retaining health care workers in rural areas have been explored in a variety of settings, there is little data outside of South Africa. As the Botswana medical school recently graduated its first class, it is important to understand students’ perceptions of factors that could influence their future career choices. The aim of this study was to explore perceptions of third- and fifth-year medical students about working in rural areas and factors that could influence their preferences of future practice.

The objectives of the study were:

1. Determine Botswana-trained third- and fifth-year medical students’ intentions to practice in rural health care settings.
2. Establish students’ perceived motivations and barriers for choosing a rural setting as future practice location.

## Research method

This study was a cross-sectional, mixed-methods design; using both a quantitative and qualitative design in the formats of a questionnaire and a semi-structured interview. Rural was defined as any location outside of the two largest population centres in Botswana namely; Gaborone, and Francistown. These two towns represent the major urban centres and are the only places where tertiary health care is available and more frequently provided by specialists. This definition was based on a definition by Couper that suggests ‘rural’ in the health care context ‘relates to the provision of health services to areas outside metropolitan centres where there is not ready access to specialist, intensive and/or high technology care and where resources, both human and material are lacking’.^14^

### Setting, study population, sampling and data collection

This study targeted all undergraduate third-year (41) and fifth-year (36) medical students at the end of or after completion of their family medicine rotations between January 2014 and August 2014. The third-year students had completed 2-week rural placements in their first and second year and one 8-week family medicine rotation. In addition to the mentioned rotations of the third years, the fifth years completed an 8-week public health rotation in year four and a second 8-week family medicine rotation in year five. Students were recruited through email, SMS messages, and phone calls. Data were collected through interviews conducted at a location and time convenient to the student. Interviews were performed at varying times between the last week of the family medicine rotation and up to 4 months after the rotation. The interviewers were University of Botswana staff who had no clinical, supervisory, or teaching interaction with the students. After obtaining informed consent, a questionnaire was used to determine the demographic characteristics, exposure to rural settings prior to medical school, and a Likert scale to assess perceptions of practicing in rural areas. The semi-structured interview was audio recorded then transcribed for analysis. It used short questions with structured probes to understand the students’ in-depth opinions about working in the rural areas, the factors that could influence their choices of practice location, and whether their rural rotations have influenced their views towards practicing in a rural setting.

### Ethical considerations

The research project was reviewed and approved by the University of Botswana, Ministry of Health in Botswana, and the University of Pennsylvania Institutional Review Boards. All interviews were voluntary and by informed consent. Data were collected by staff that did not have teaching or clinical interactions with the students.

### Data analysis

The data were analysed using descriptive statistics in SPSS 22. Descriptive statistics were used to summarise the demographic and questionnaire data. The recorded interviews were transcribed, coded independently by two persons (GT and TAM) with reconciliation of coding discrepancies, and then analysed for common themes and subthemes from the data. The thematic framework used for the analysis was based on the literature suggesting that student background, training, and professional and health system issues, will affect students’ perceptions of rural practice. The questionnaire data on perceptions of rural care was compared with the responses in the interview. The Qualitative analysis used Atlas.ti 7 software.

## Results

Interviews were conducted with 35 (45%) of 77 possible third- and fifth-year students. The average age was 23.4 (SE ± 0.229) years and 17 (49%) were male. Demographics and characteristics of those interviewed are summarised in Table 1. Before entering medical school 63% (22) had lived in a rural setting for more than five years which we refer to as a having a rural background. Differences were not seen in the questionnaire responses or types of comments in the interviews between the fifth years and third years. Thus the themes and responses and discussion have combined the two-year groups.

**TABLE 1:** Demographics and characteristics of the participants.

Variable	Category	*N*	Proportion(%) ± s.e.	Mean ± s.e.
Age (years)	-	35	-	23.4 ± 0.229
Gender	Male	17	49 ± 8.45	-
	Female	18	51 ± 8.45	-
Rural background	No one	9	26	-
	< 5 years	4	11	-
	5–10 years	7	20	-
	11–15 years	4	11	-
	16–20 years	10	29	-
	20 years	1	3	-
Year in medical school	Year 3	20	57 ± 8.37	-
	Year 5	15	43 ± 8.37	-
Length of rural exposure (weeks) during medical school	5–10 weeks	18	51 ± 8.45	-
	11–15 weeks	2	6 ± 4.01	-
	16–20 weeks	4	11 ± 5.29	-
	20 weeks	11	31 ± 7.81	-
Preferred future placement	Rural	3	9 ± 4.84	
	Urban	32	91 ± 4.84	-
Preferred future employer	Ministry of Health	9	26 ± 7.41	-
	University	14	40 ± 8.28	-
	Private and non-governmental organisation	4	12 ± 5.17	-
	Mine	7	20 ± 6.76	-
Preferred type of facility	Tertiary/Academic	23	66 ± 8.01	-
	District	5	14 ± 5.87	-
	Primary	1	2 ± 2.37	-
	Psychiatric	3	9 ± 4.84	-
	Private & Mine hospital	3	9 ± 4.84	-

*Source*: Authors own work

### Student future practice preferences

The majority 33/35 (96%) of respondents desire to practice in Botswana and 32/35 (91%) want to practice in an urban area. However, 31% said it was likely/very likely that they would practice in a rural area. In the interviews several students did comment that the rural rotations showed them that practicing in a rural area is not as difficult as previously perceived.

A third-year student:

‘it made me realize it won’t be the worst to practice outside an urban area.’ (participant 05 02 301)

A fifth-year student:

‘because before I came to the rural setting I had absolutely no intention or no desire to work in a rural area but now I am more willing to do it, but I’m still, it’s not like my first choice or something.’ (participant 02 01 501)

In addition, a couple students said they are now considering family medicine or public health because of these rotations. The public sector, either directly in the ministry of health facilities, or with the university, is the preferred future employer (68%). Consistent with the literature students identified personal and professional issues that currently influence their perceptions on rural practice and perceived future practice location.

### Professional development issues

Only 6% of the interviewees felt that rural practice provided opportunities for career advancement whereas 69% disagreed/strongly disagreed with this statement. Two-thirds of the students also perceived that working in the rural areas leads to professional isolation. These issues were raised repeatedly in the interviews. The perception that there is career stagnation, lack of learning opportunities, and professional isolation were recurring themes. For example, in response to being asked what are barriers to practicing in a rural area?

A fifth-year student noted:

‘if there is no way I could enhance my career in a rural setting, like there is no training or lecturing going on.’ (participant 02 02 501)

Another fifth-year stated:

‘I’m not getting support … like we don’t go for trainings or anything, it is like you’re just left there alone in a rural area.’ (participant 02 02 502)

‘And a third year said rural practice is *place is stagnation.*’(participant 04 02 301)

The students did have some positive perceptions of rural practice. Seventy-seven percent agree that people in the rural areas are friendly and 52% believe that rural staff are more supportive of each other. These findings were supported by interview comments.

For example:

‘I feel the people there work together. There is more of [an attitude of] like…if we [are] going to do [this] let’s do it together’ reported a fifth year student. (participant 05 02 502)

Although in the survey 74% report that practicing in a rural area allows for clinical practice autonomy and 49% see rural practice has the opportunity to practice a variety of skills, no students mentioned practice autonomy or the opportunity to practice a variety of skills as positives of rural care during the interviews. Thus, it seems these factors were not perceived as strong influencing issues on practice location for the students.

Students did mention several ways to decrease the professional isolation. They noted the need to feel supported by the system. Several students noted that if they had easy access to specialist support and received feedback from specialists on referred patients, rural practice would be more attractive. They saw this type of support as a way to encourage learning and prevent the feeling of stagnation while practicing in a rural environment. For example on being asked what, if changed, might attract one to a rural practice.

A fifth-year said:

‘support from the bigger hospitals when it comes to maybe referring or getting advise on the way forward for the patient.’ (participant 02 02 502)

Another fifth-year said:

‘even if say, just specialists, maybe once in a while you get people from, …specialists here and there to come and give a lecture on a certain topic here and there…talking to all the doctors,… addressing all the doctors, teaching all the doctors and even skills…’ (participant 02 01 502)

They also suggested that making sure that these areas had good access to the internet would be beneficial as they would allow for learning and access to resources.

A third-year said:

‘Would want to have any material that I would need to make it easier if I need to access information.’ (participant 02 01 301)

A fifth-year noted a barrier to rural practice was:

‘(No) access to like internet, and libraries, that might also be a problem.’ (participant 02 02 501)

Although a couple students said that the facilities in rural areas were new and equipped, the majority noted that they were not fully functional and this would be a barrier to practicing in a rural area.

A third-year said:

‘some maybe investigation that you want to do, there won’t be those as compared to like urban areas, some labs that you want to use, some technologies that you want to use maybe you will be limited when you are in rural areas.’ (participant 03 01 301)

And a fifth-year noted:

‘even small things like glucose sticks, things that would make a difference, but if you are working in a rural area where there is no maybe IV fluids, there are no glucose sticks which I think they are very minimal staff to have in a health facility.’ (participant 02 02 502)

### Personal issues

Those with a rural background were more likely to practice in a rural area if it was near their home towns. None of the students from an urban background suggested that proximity to home as a barrier or a reason to practice in a rural area. Many noted that a lack of recreation was a barrier to staying in rural areas. Seventy-one percent agreed with the statement that there is a lack of recreation in rural areas.

For example a third-year student noted:

‘There are no entertainment facilities and no place to go; the cinema, no parks and transport is really difficult to get.’ (participant 05 01 302)

Many did mention monetary compensation as a way to attract them to rural practice but the issues of professional development and functional facilities were mentioned as frequently. In addition, lack of basic infrastructure was also a barrier, for example, a lack of good schools or a regular water supply. However, this was less prominent than the issues of recreation, professional development, and functional facilities.

## Discussion

This study adds to the growing body of literature on African medical students’ perceptions of rural practice and career intentions. Botswana students, similar to findings in other countries, prefer urban practice. We found a high level of rural background prior to medical education. This background does seem to influence the perceptions of students in that those from a rural background were more likely to perceive the attraction of living near a home village or town but the pull of urban practice dominates. Our small sample size with a high level of rural background precluded further assessment of statistical associations between rural upbringing and practice preferences. The literature suggests that those from a rural background are more likely to return to rural areas.^5,9^ However, the evidence suggests that this alone is not enough to ensure a return to rural areas and this study supports that theory.^5,8,15,16^

Botswana students do want to do specialty training outside Botswana but most plan to return. This is more similar to findings of South African students than students from East or West Africa where a higher percentage indicate the desire to emigrate.^7,17,18^ The majority of Botswana interviewed students indicate a desire to do specialty training on the African continent with South Africa as the first choice. However, the local trend previously for medical education is that only a small fraction of students doing undergraduate medical education abroad return home.^13^ Time will tell if doing the undergraduate training locally but post graduate specialisation overseas will reverse the loss of doctors to other countries where they trained. Because of limitations with the sample size, factors such as gender, socioeconomic status, and length of previous rural exposure could not be correlated with planned practice.

The literature suggests that strong rural exposure during medical education can have a positive influence on students’ attitudes towards rural practice.^5,19^ Despite the efforts of the medical school to incorporate rural education throughout the undergraduate curricula most students felt it did not influence future practice choices. As previously noted we did not see differences in responses between the third years and fifth years suggesting the length of exposure also did not influence the students’ perceptions. This supports the concept that rural exposure during training is not enough to entice students to practice in rural areas. However there were a couple of students who noted that the experiences showed them that rural practice is not as ‘bad’ as they had previously perceived. Thus, it cannot be said that rural exposure in training had no influence and it may be beneficial if a couple of students out of a small class of 77 a year consider rural practice or it lowers resistance to such postings. It is not just quantity but the quality of the rural experience that can be influential as supported by an Australian study, which stresses the role of the quality of education in accepting rural internships.^20^ Thus, it is possible that strengthening these rotations may lead to the possibility of influencing students’ perceptions of rural practice positively. These issues were explored in the interviews and are reported elsewhere. However, in as much as rural training has been reported to have a promotional influence on rural career intentions, competition between choosing a rural career and concurrent personal life choices will remain.^21^

The professional factors highlighted by this study are similar to other studies.^22^ They highlight the need to address professional growth and support for rural practitioners in order to increase the attraction of this type of practice. Although students mentioned the potential of monetary incentives for rural practice this seemed at best equal to the issues of professional stagnation and few learning opportunities. This is supported by the literature, that monetary incentives have added benefit but alone are not enough to attract professionals to rural practice.^22,23^ However, a limitation of this study was the young average age of the students which might influence their perceptions of prioritising education and career over monetary gain as they don’t yet have families to support.

### Limitations

There are several limitations to this study. It is specific to the Botswana context. However, the issues raised are similar to those found in other settings. It is possible that the opinions of those students not interviewed would be different. However, the themes of those interviewed were consistent and similar to observations in other studies. Therefore it is not likely that interviewing more students would have changed the findings. The average age of the students is young and they are still in the formative years of their education. It is possible that the priority they placed on educational opportunities in rural practice and career advancement may change as they progress in their careers. It will be important to see whether the views are maintained by those further along in training and careers.

## Conclusion

In conclusion, many Botswana medical students come from a rural background prior to entering medical school. The exposure they get during the undergraduate training so far seems to have minor influence on career choices but this needs to be further explored to assess the quality of the exposure. They have identified factors of professional growth, remuneration, and support by the health system that, if addressed, may make rural practice more enticing.
